# Better Writing in Scientific Publications Builds Reader Confidence and Understanding

**DOI:** 10.3389/fpsyg.2021.714321

**Published:** 2021-08-27

**Authors:** Ren Ryba, Zoë A. Doubleday, Matthew J. Dry, Carolyn Semmler, Sean D. Connell

**Affiliations:** ^1^Southern Seas Ecology Laboratories, School of Biological Sciences, The University of Adelaide, Adelaide, SA, Australia; ^2^Future Industries Institute, The University of South Australia, Mawson Lakes, SA, Australia; ^3^School of Psychology, The University of Adelaide, Adelaide, SA, Australia

**Keywords:** accessibility, confidence, interdisciplinarity, readability, scientific writing, traditional style, understanding

## Abstract

Scientific publications are the building blocks of discovery and collaboration, but their impact is limited by the style in which they are traditionally written. Recently, many authors have called for a switch to an engaging, accessible writing style. Here, we experimentally test how readers respond to such a style. We hypothesized that scientific abstracts written in a more accessible style would improve readers’ reported readability and confidence as well as their understanding, assessed using multiple-choice questions on the content. We created a series of scientific abstracts, corresponding to real publications on three scientific topics at four levels of difficulty—varying from the difficult, traditional style to an engaging, accessible style. We gave these abstracts to a team of readers consisting of 170 third-year undergraduate students. Then, we posed questions to measure the readers’ readability, confidence, and understanding with the content. The scientific abstracts written in a more accessible style resulted in higher readability, understanding, and confidence. These findings demonstrate that rethinking the way we communicate our science may empower a more collaborative and diverse industry.

## Introduction

Scientists belong to a global culture, and we are transforming our sector into one that is diverse, inclusive and equitable. We are moving beyond narrow fields and collaborating across disciplines to address the world’s biggest problems. And we are making science relevant and useful to medical practitioners, policy makers, and innovators to name just a few. Unfortunately, however, stubborn gaps persist in this age of diversity. Women and non-binary and transgender people remain underrepresented in science and senior positions (Beemyn, 2015; Leslie et al., 2015), scientists still have trouble communicating across disciplines (Winowiecki et al., 2011), and the uptake of science by the general public remains limited and skewed (Suleski and Ibaraki, 2010; Herrando-Perez et al., 2019).

Scientific publications are the building blocks of knowledge and collaboration. Understanding them builds a reader’s confidence—both within a scientific discipline, and in their own ability to explore new frontiers across disciplines. Building confidence could also help reduce persistent gender gaps in science (Leslie et al., 2015), reduce language barriers to people speaking English as a second language (Hanauer and Englander, 2011), and assist people with learning disabilities like dyslexia, who also need the energy and determination that comes from the confidence of understanding (Vellutino et al., 2004).

More accessible writing can help. Science has the opportunity to move beyond the traditional scientific writing style (Pinker, 2015; Doubleday and Connell, 2017; Ryba et al., 2019). The traditional writing style strives for objectivity and distance from the subject, but also remains dense, formulaic and difficult to read. Similar to a pay-wall, it acts as a linguistic-wall. In a time of intense policy debate over open access publishing (Tofield, 2019), we should also consider policies that build reader confidence and linguistic access to science (Jeschke et al., 2019).

Scientific writing is explored in countless guides and manuals (e.g., Montgomery, 2003; Sword, 2012; Pinker, 2015). These books differ on many points, but they all agree that the essential characteristic of scientific writing is clarity (Heard, 2014). However, clear scientific papers are rare. One study on over 7,00,000 scientific papers published in life, medical, social, and multidisciplinary sciences between 1881 and 2015 documented a rise in technical jargon; in fact, over one fifth of scientific abstracts were written at a level that even a college graduate would struggle to comprehend (Plavén-Sigray et al., 2017). This study was successfully replicated using data on around 28,000 abstracts published in management research between 1991 and 2019 (Graf-Vlachy, 2021). A similar study, which assessed around 12 million scientific abstracts from a multidisciplinary collection of publications from 1900 to 2018, also found that papers are becoming more difficult to read (Vergoulis et al., 2019). Also, an analysis of 24 million article titles and 18 million abstracts published in health and medicine between 1950 and 2019 found an increasing use of obscure acronyms (Barnett and Doubleday, 2020). The rise in jargon and reading difficulty may reflect the trend toward writing styles that are specific to, and isolated within, single disciplines (Ngai et al., 2018).

This increasing impenetrability of science presents a paradox: peer-reviewed publications are the universal currency for creating and communicating scientific knowledge, but the language is increasingly ill-suited to this purpose (Doubleday and Connell, 2017). Some solutions have been offered, such as lay-reader summaries for clinical findings in medical journals (Kuehne and Olden, 2015). However, inaccessible language threatens the accessibility and reproducibility of new scientific findings (Plavén-Sigray et al., 2017). Difficult-to-read papers can compromise readers’ ability to understand information and can threaten their support for new ideas (Bullock et al., 2019). Furthermore, accessible information is critical for making decisions that depend on coordinating knowledge from multiple fields (Millgram, 2015).

To address this problem, many scientists now advocate for an alternative: a scientific writing style that is clear and accessible. Doubleday and Connell (2017) promote scientific language that is clear, accessible, and even inspiring, while maintaining the objectivity critical to science. Sand Jensen (2007) argues that accessible language can attract scientists, unify our understanding, and promote timely debate and discussion. Hartley et al. (2002) write that clear scientific writing can encourage exchanges between researchers, practitioners, and the general public, and that making science accessible is actually the duty of taxpayer-supported scientists. Empirical studies also provide support for accessible scientific writing, with several studies finding correlations between writing style and impact (Weinberger et al., 2015; Hillier et al., 2016; Ryba et al., 2019).

Is this boost in influence a result of how readers respond to more accessible writing? Indeed, in educational research it has long been established that more readable writing is more easily understood (Dale and Chall, 1949), which, in turn, boosts a reader’s confidence in the message (Galbraith and Alexander, 2005).

So, this study aimed to measure how more accessible scientific publications stimulate reader understanding and confidence. Specifically, we tested the hypotheses that better writing in scientific publications can enhance readability, reader confidence, and reader understanding.

## Materials and Methods

### Experimental Manipulation

To test the effect of reading style on readability, confidence, and understanding, we performed an experimental manipulation with randomized participants. We created a series of scientific abstracts corresponding to real, recent, peer-reviewed publications in three research topics: social anxiety (health sciences), solar cells (physics), and populist politics (social sciences). We used abstracts as a proxy for full articles, as abstracts follow a relatively consistent structure and reflect the content and writing style of the full publications (Plavén-Sigray et al., 2017; Ryba et al., 2019).

Each abstract was manipulated to create four variants, ranging in readability from very difficult to very easy. We achieved this variation in difficulty by including different systematic combinations of nine measurable components known to affect writing style (Table 1). We selected these components as a measure of clear, creative writing style following a recent observational study, which derived them from findings in psychology, English, and science communication literature (Ryba et al., 2019). That study used a set of 11 measurable components, and here we adopted the nine that can be objectively manipulated when constructing new versions of the same abstract. Table 1 contains definitions for each component, and further details and references are provided in that study (Ryba et al., 2019). The nine components signal either clear and inspiring writing, as with signposting and setting, or awkward and obscure prose that worsens the cognitive load on the reader, as with acronyms or extra words that dilute the central message (Pinker, 2015; Ryba et al., 2019). (We excluded “consistent language” and “parallel phrasing” from Ryba et al. (2019), as those two components cannot be readily added to abstracts without altering the flow of content and logic).

**TABLE 1:** The combination of writing components that we used to generate new abstracts of a range of qualities (see Ryba et al., 2019 for further details on each writing component).

	**Setting**	**Narrator**	**Punctuation**	**Conjunctions**	**Signposts**	**Noun chunks**	**Acronyms**	**Hedges**	**Words**
**Definition**	**Does the abstract explicitly mention a time or place?**	**Does the abstract use “we” or “I”?**	**Number of colons, dashes and semicolons between words**	**Number of conjunctions used to link ideas by cause-and-effect, contrast, or order**	**Number of adverbs defining order (e.g., *lastly*) at the start of a sentence or idea**	**Number of groups of three or more consecutive nouns**	**Number of acronyms**	**Number of words that dampen the confidence of claims (e.g., *potentially*)**	**Number of words in total**
Easiest	Yes	Yes	2	1	2	0	0	0	110
Easy	Yes	No	2	1	2	2	3	2	160
Hard	No	No	0	0	0	4	6	4	160
Hardest	No	No	0	0	0	6	15	4	230

*The first five components make for clear, engaging writing, while the latter four are typical of the traditional style and make for a dull, confusing read (Ryba et al., 2019).*

We took care to keep the range of writing components within the range that we have recorded from real-world publications. We also retained all content and logical flow across the different writing styles, meaning that the level of detail is the same across all styles for a given topic. This way, we generated writing styles that ranged from the traditional to the accessible, with two intermediate styles, while crossing each of the four styles orthogonally across the three topics. An example of the writing styles is given in Supplementary Table 1.

### Participants

We then asked teams of volunteer readers to read the abstracts. To obtain readers who were consistent in scientific background and age group, we approached four classes of third-year undergraduate science students (173 students in total). We offered all participants the opportunity to participate, and participants were permitted to opt out either verbally or by selecting the appropriate option on the consent form.

Each participant completed up to three readings. For a given participant, the topics and difficulty levels were randomly assigned using a random number generator, with the condition that the readings be of different topics ensuring that students could not learn any abstract content between readings. Participants were not made aware that there were multiple levels of difficulty.

**FIGURE 1:**
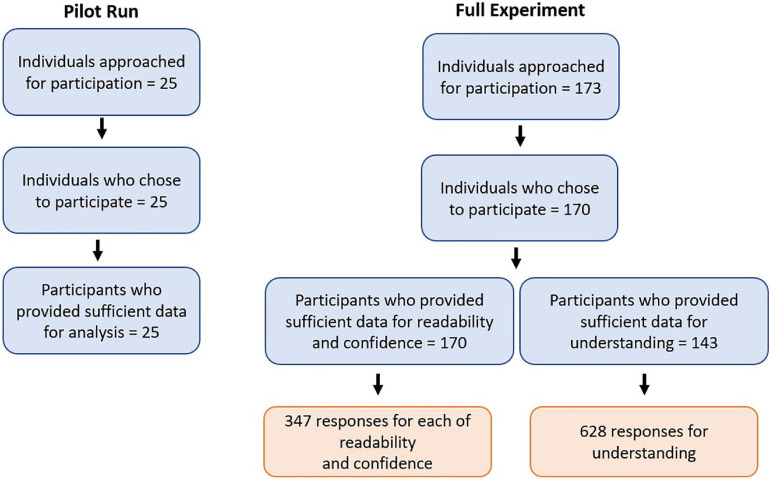
Flow of participants through the pilot run and the full experiment.

We approached 173 individuals for participation in the full experiment, 170 of whom began the experiment. Thus, the data analysis included responses from 170 participants, meaning that we analyzed 347 responses for each of readability and confidence and 628 responses for understanding. We excluded the models from any observation with incomplete data for each model variable. The detailed participant flow is charted in Figure 1. 87.1% of participants were native English speakers.

### Measures

We asked our readers to read each abstract. Readers then answered two questions on a scale from one to five: “how easy was this abstract to read?” (readability) and “how confident are you that you understood the information present in the abstract?” (confidence). Each reader was then independently tested with two multiple-choice questions about the content of the abstract (understanding). Those multiple-choice questions were “Why was this research conducted?” and “What is the take-home message of this research?”; the multiple-choice answers differed depending on the topic (example in Supplementary Table 1). We acknowledge that the use of 1–2 questions for each variable is a limitation; we chose to ask fewer questions to allow us to obtain multiple readings from each participant, as the statistical power of the study was a priority. Using this approach, we could determine how writing style affected readability, confidence, and understanding, while also accounting for topic.

### Procedure

Ethics approval was obtained from the School of Psychology Human Research Ethics Committee at the University of Adelaide (approval number H-2019/18).

Each reader was given 5 min to read the abstract and answer the questions, although no reader used the entire duration. In total, we received 347 valid responses for each of readability and confidence, and 628 valid responses for understanding.

The experiment was performed during normal teaching hours in March 2019 and in March 2021. The experimental procedure differed between the 2 years only in that participants completed up to two readings each in 2019, and up to three in 2021. Prior to the experiment, we conducted a pilot run, used to estimate the effect size and calculate an appropriate sample size for the full study. We chose our sample size as 300 observations for each of reading and confidence and 600 for understanding, given the effect sizes for each response variable observed in our pilot study, yields a power above 0.99 at the significance level of 0.05. This calculation was performed using the R package *simr* (Green and MacLeod, 2016), which uses Monte Carlo simulations to perform power analysis for generalized linear mixed models. Each session lasted approximately 30 min.

### Data Analysis

We expressed each of the four levels of reading difficulty as a continuous variable. This continuous variable was calculated using a method from previous research on readability (Hillier et al., 2016; Ryba et al., 2019). Firstly, we performed principal component analysis to reduce the nine writing components in each level of difficulty to a series of uncorrelated axes. Then, we identified which of these axes correlated positively with the “good” writing components and negatively with the “bad” components. This axis (the first—which also explained most of the variation in the writing components) was used as a continuous variable representing reading difficulty.

Mean confidence, readability, and understanding for each level of difficulty, averaged across all topics, is provided in Table 2. For readability and confidence, we rescaled the predicted values to percentages (1.0 = 0; 5.0 = 100%). For understanding, we converted each multiple-choice question to a binary response (0 = incorrect; 1 = correct). We then rescaled the probabilities of obtaining a correct response to percentages.

**TABLE 2:** The mean readability, reader confidence, and reader understanding observed in the experiment at each level of difficulty as generated from the responses of participants.

	**Readability**	**Confidence**	**Understanding**
Easiest	66.29 (29.94)	62.36 (29.71)	57.87 (49.52)
Easy	58.51 (28.53)	57.98 (29.38)	39.47 (49.04)
Hard	52.31 (26.27)	55.03 (26.01)	40.79 (49.31)
Hardest	44.44 (29.44)	48.78 (30.56)	47.95 (50.13)

*Standard deviations are given in brackets.*

To estimate the effect of reading difficulty on confidence and readability, we generated linear mixed models. We used linear mixed models to account for the correlation that arises from taking multiple measurements from each participant (Jostins et al., 2012). We included terms for fixed effects of reading difficulty and topic, and for random effects of participant. This random effect captures the effect of specific participants, which might include characteristics such as reading strength and familiarity with the topic, on the response variables. We allowed for random effects in the intercept, but not the slope, as there were only a few measurements from each participant. For the effect of reading difficulty on understanding, the response was binary. So, we generated a generalized linear mixed model using the binomial family with a logit link function. All data analysis was performed in R (R Core Team, 2020). Models were generated using the R package *lme4* (Bates et al., 2015) and tabulated using the package *sjPlot* (Lüdecke, 2020). We performed significance tests on the coefficients using the package *lmerTest*, which uses Satterthwaite’s degrees of freedom method (Kuznetsova et al., 2017).

## Results

**FIGURE 2:**
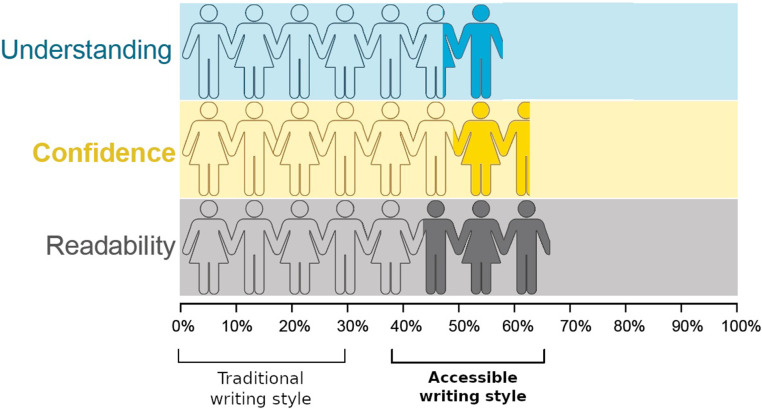
Accessible writing boosts readability (*p* < 0.001), reader understanding (*p* = 0.027), and reader confidence (*p* = 0.001). The stick figures represent the proportional gain in readability, confidence and understanding by writing in the most accessible style (filled figures), compared to the most traditional style of writing (hollow figures). Each stick figure represents 5%.

So, what does better writing mean for the reader? When we presented readers with abstracts written in the most traditional scientific writing style, mean readability was 44.4% (SD: 29.4%). In contrast, when we presented readers with abstracts written in an accessible, engaging style, mean readability was 66.3% (SD: 29.9%) (Figure 2). A similar effect was found for understanding, which resulted in a mean of 47.9% (SD: 50.1%) for the traditional style and 57.9% (SD: 49.5%) for the accessible style. Likewise, confidence ratings were 44.1% (SD: 23.7%) and 58.3% (SD: 32.3%), respectively, for the traditional and accessible styles, respectively. The mean scores of readability and confidence increased consistently from the most difficult to the easiest writing styles.

The models (Table 3) revealed statistically significant effects of writing style on readability, understanding, and confidence. The differences between each level of writing style, after accounting for the effects of topic and reader, are revealed by the effect sizes. For readability, the slope was 2.91 (confidence interval: 1.79–4.03), indicating that a one-unit increase in writing style was associated with a 2.91% increase in readability. For understanding, the odds ratio was 1.08 (1.01–1.15), indicating that a one-unit increase in writing style was associated with a 1.08-fold increase in odds of successful understanding. For confidence, the slope was 1.87 (0.75–2.99), indicating that a one-unit increase was associated with a 1.87% increase in confidence. As a continuous variable, writing style spanned approximately 5 units; as such, one can readily see how moving from the most difficult style to the most accessible style can boost readability, understanding, and confidence quite substantially. Likewise, the effect of topic was statistically significant on readability, understanding, and confidence. Alternative models found no evidence of an interaction between topic and difficulty, and no evidence of an effect of English as a second language (Supplementary Table 2).

**TABLE 3:** Model outputs for the effect of reading difficulty on readability, reader confidence, and reader understanding.

	**Readability**				**Confidence**				**Understanding**			
**Predictors**	**Estimates**	**CI**	***p***	**df**	**Estimates**	**CI**	***p***	**df**	**Odds ratios**	**CI**	***p***	**df**
(Intercept)	71.87	67.39–76.35	<0.001	341.00	0.89	66.30–75.47	<0.001	341.00	0.85	0.65–1.11	0.234	623.00
Difficulty	2.91	1.79–4.03	<0.001	341.00	1.87	0.75–2.99	0.001	341.00	1.08	1.01–1.15	**0.027**	623.00
Topic—populism	−14.24	−20.23 to −8.25	<0.001	341.00	−15.19	−21.05 to −9.33	<0.001	341.00	1.81	1.24–2.64	0.002	623.00
Topic—solar cells	−32.12	−38.92 to −25.32	**<0.001**	341.00	−29.69	−36.29 to −23.10	**<0.001**	341.00	0.70	0.46–1.05	0.087	623.00
**Random Effects**												
σ^2^	593.71				544.76				3.29			
τ_00_	73.97 _participant_ID_				160.45 _participant_ID_				0.05 _participant_ID_			
ICC	0.11				0.23				0.02			
N	170 _participant_ID_				170 _participant_ID_				143 _participant_ID_			
Observations	347				347				628			
Marginal R^2^/Conditional R^2^	0.244/0.327				0.186/0.372				0.053/0.068			

*CI, confidence interval; df, degrees of freedom.*

## Discussion

In this study, we sought to measure how accessible scientific writing can stimulate reader understanding and confidence. The findings reveal that writing in a more accessible style can enhance readability of and confidence with scientific publications, as well as understanding of the content. This study extends previous research on readability and confidence (e.g., Galbraith and Alexander, 2005) to the context of scientific publications. Furthermore, this study provides evidence that more readable writing is more easily understood (Dale and Chall, 1949). So, the evidence supports the hypotheses that better writing in scientific publications can enhance readability, reader understanding, and reader confidence.

The magnitude of the effect is quite striking. The finding that mean reader confidence, for example, was 62% for the accessible style (compared to 49% for the traditional style) illustrates that the way publications are written can cause readers to be far more confident when digesting scientific advances. Indeed, the research described in the abstracts was complex, as scientific advances tend to be. With that in mind, it is easy to recognize the valuable opportunity before us.

Just as we aim to hone our science, we can also further that goal by honing our writing—in essence, writing with the comprehension of the reader in mind (Doubleday et al., 2017). These findings indicate that we all have a concrete opportunity to boost the confidence of our readers and promote access to science through simple stylistic choices. Specifically, a setting can help ground the research in time and space; first-person narration can enhance clarity; punctuation marks can help guide the reader’s attention; conjunctions can help to link complex ideas; and the judicious use of signposts can help order and structure a piece of writing. Likewise, avoiding acronyms can ease the reader’s mental load; breaking up noun chunks can help the reader digest ideas; and hedging only where necessary can help emphasize the message (Montgomery, 2003; Sand Jensen, 2007; Sword, 2012; Lindsay, 2013; Pinker, 2015; Weinberger et al., 2015; Hillier et al., 2016; Ryba et al., 2019; Barnett and Doubleday, 2020).

Future research can complement the subjective measures we report here by collecting further measures. In particular, since readability, confidence and understanding were collected from self-reported questions, objective measures could shine further light on how readers respond to different abstracts. For example, objective measures could be calculated from the time that readers take to read texts (Maksymski et al., 2015); from existing metrics of writing style such as the Flesch Reading Ease (Flesch, 1948) or the Dale–Chall Readability Formula (Chall and Dale, 1995); and from more detailed tests on abstract content. Also, subjective measures could enable participants to report their attitudes and feelings toward the content (Olney et al., 1991). These measures can enable future researchers to delve deeper into how readers respond to better scientific writing. Furthermore, this study detected no difference of lower readability, understanding or confidence for participants for whom English as a second language. These readers likely experience a greater burden in reading and writing research articles (Hanauer and Englander, 2011), although this study and sample was not designed to test that question. A future study could replicate our analysis using a larger sample of readers for whom English is a second language; this would reveal how the findings here could reduce the burden on those readers.

Evidence can be a powerful motivator for the scientific community to revise the way they communicate science. This study signals two compelling avenues for research inquiry. First, it would be progressive to recognize the relative extent to which different disciplines benefit from writing in a more accessible style. Where readability is improved, to what extent does greater reader confidence and understanding also improve a scientist’s willingness to make connections between concepts, methods and interpretations? It is likely that progress, particularly in interdisciplinary research, will be more rapid where those searching for solutions have the confidence to engage with alternate approaches (e.g., disciplines) (Brandt et al., 2013).

 Second, we may benefit from recognizing how more accessible writing benefits the spectrum of expertise among science readers. Our study involved a sample of one particular audience of scientific publications—specifically, third-year undergraduate students. Students represent one group of readers of scientific papers, and so replicating the study with samples from different audiences (e.g., post-doctorate researchers, senior scientists, policymakers) would enable us to see whether the findings are generalizable to all readers (Zahedi et al., 2014; Mohammadi et al., 2015). If society is to perceive science as relevant to their well-being, then scientific uptake not only relies on science literacy (Ryder, 2001), but also the readability of science. Whilst there is a variety of science forums, namely those for members of the public and other non-expert groups, it would be insightful to research whether greater readability results in greater inclusion of readers from diverse groups.

Securing the boost in readability and confidence would mean updating the practice and teaching of scientific writing. Journal policies and university courses have, traditionally, promoted a writing style that is distant and formulaic (Doubleday and Connell, 2017). But there is a wiser way. As we have found here, a boost in confidence can be made through better writing. Journal editors and teaching staff who encourage readable, accessible writing may help the new generation of scientists explore new topics and collaborate more easily across disciplines. Indeed, recent advances in this area have stimulated discussion on writing policy in scientific journals (e.g., Allen, 2019; Chaffey, 2019; Sayer, 2019). And there are published style guides that give instruction on writing scientific publications in an inspired way (e.g., Montgomery, 2003; Sword, 2012; Pinker, 2015). Our evidence suggests that the widespread adoption of these style guides could boost the confidence of people who read scientific publications.

By cultivating reader confidence in understanding, we could empower a more diverse readership, making science both more inclusive and connected. Developing our writing style to one that is more accessible and inclusive requires a bold and proactive approach. We are trying to open up access from behind the pay-wall through bold initiatives, such as Plan S which aims to ensure that publicly funded research is published in publicly accessible journals (Tofield, 2019); likewise, we also need to open up access from behind the linguistic-wall (Doubleday and Connell, 2017). We believe that scientists who write with the reader in mind will empower their readers to build confidence and challenge new boundaries. With a growing diversity of bright minds in research, we will be in the best position to develop major advances in the twenty-first century.

## Data Availability Statement

The raw data supporting the conclusions of this article will be made available by the authors, without undue reservation.

## Ethics Statement

The studies involving human participants were reviewed and approved by H-2019/18. The patients/participants provided their written informed consent to participate in this study.

## Author Contributions

RR and SC performed investigation. RR performed data curation and formal analysis and wrote original draft. All authors contributed to review, editing, conceptualization, and methodology.

## Conflict of Interest

The authors declare that the research was conducted in the absence of any commercial or financial relationships that could be construed as a potential conflict of interest.

## Publisher’s Note

All claims expressed in this article are solely those of the authors and do not necessarily represent those of their affiliated organizations, or those of the publisher, the editors and the reviewers. Any product that may be evaluated in this article, or claim that may be made by its manufacturer, is not guaranteed or endorsed by the publisher.
