# Supplementary material for: Better Writing in Scientific Publications Builds Reader Confidence and Understanding
Source: Front Psychol. 2021 Aug 27;12:714321. doi: 10.3389/fpsyg.2021.714321 (PMC8430246; doi:10.3389/fpsyg.2021.714321)
Supplement: Supplementary file 1 [file Data_Sheet_1.docx]

Supplementary Material

**Table S1**. An example of the writing styles used in this study, from easiest (i.e, most accessible) to hardest (i.e., most traditional and impenetrable). This example shows the experimental treatments for the topic of populism. The questions used to assess readability, confidence, and understanding are also shown.

| Easiest | Over the past decade, the world has witnessed a rise in populism. How is social media related? In this study, we explore the ways that European populists use social media. Firstly, we derived five elements of populism from existing literature. Then, we performed a qualitative text analysis – this showed that a broad range of politicians advocate for the people on social media. Left-wing populists preferred attacks on economic elites; conversely, right-wing populists preferred attacks on media elites and ostracism of others. Our research demonstrates that social media gives populists the freedom to articulate populist ideology. We must safeguard democracy from this harmful ideology. |
| --- | --- |
| Easier | Over the past decade, the world has witnessed a rise in populism. In this study, the way that politicians use social media (SM) for populist purposes was explored. From existing literature, five populism media elements were derived. These were: emphasizing the sovereignty of the people, advocating for the people, attacking the elite, ostracizing others, and glorifying the community. Then, a qualitative text analysis was performed. Populism elements were found across countries, parties, and politician status levels. The analysis showed that a broad range of politicians advocate for the people on SM. However, populists differed in some behaviours on SM depending on their position on the political spectrum. Left-wing populists largely preferred attacks on economic elites; right-wing populists preferred attacks on media elites and ostracism of others. Taken together, the research demonstrates that SM potentially gives populists the freedom to articulate populist ideology. The work identifies ways to conceptualize and measure populism, which will be useful for future research. |
| Harder | There has been a rise in populism. In this study, the way that politicians use social media (SM) for populist purposes was explored. From existing literature, five populism media elements (PMEs) were derived. PMEs were emphasizing the sovereignty of the people, advocating for the people, attacking the elite, ostracizing others, and glorifying the community. A qualitative text analysis (QTA) was performed. PMEs were found across countries, parties, and politician status levels. The QTA showed that a broad range of politicians may advocate for the people on SM. There were ideology spectrum differences in populist behaviours on SM. Left-wing populists largely preferred attacks on economic elites. Right-wing populists generally preferred attacks on media elites and ostracism of others. The research demonstrates that SM potentially gives populists the freedom to articulate populist ideology. Populism conceptualization methods and populism measures may be useful for future research. |
| Hardest | There has been a rise in political populism (PP). PP has been well-researched in public politician statements and the mass media. In this study, the way that politicians use social media (SM) for PP purposes was explored. Numerous scholars have analyzed politicians’ use of various websites. They either have ignored the concept of populism or alluded to it only briefly. From existing literature, five PP media elements (MEs) were derived. MEs were emphasizing the sovereignty of the people, advocating for the people, attacking the elite, ostracizing others, and glorifying the community. A qualitative text analysis (QTA) was performed. PP manifests itself in a fragmented form on SM. MEs were found across countries, parties, and politician status levels. The QTA showed that a broad range of politicians may advocate for the people on SM. There were ideology spectrum differences in PP behaviours on SM. Left-wing populists largely preferred attacks on economic elites. Right-wing populists generally preferred attacks on media elites and ostracism of others. The paper provides an in-depth analysis of PP on SM. SM potentially gives populists the freedom to articulate PP ideology. Populism conceptualization methods and measures may be useful for future research. |
| Question to measure readability | **On a scale of 1 to 5, how easy was this abstract to read?**  1 2 3 4 5  Very difficult Very easy to read |
| Question to measure confidence | **On a scale of 1 to 5, how confident are you that you understood the information present in the abstract?**  1 2 3 4 5  Not confident at all Very confident |
| Multiple-choice questions to measure understanding | **Why was this research conducted?**   1. To derive five populism media elements from existing literature 2. To analyse politicians’ use of various websites 3. To produce populism conceptualization methods and measures 4. To see how social media is related to populism [correct]   **What is the take-home message of this research?**   1. The populism conceptualization methods and measures are important for future research 2. Populism behaviour on social media depends on the ideology spectrum 3. Social media enables politicians to use populist ideology [correct] 4. Populism exists across many countries, parties, and politician statuses |

**Table S2**. Alternative models for readability and confidence. These models consider an interaction between difficulty number and topic (first two models) or the effect of English as a second language (latter two models). The models show statistically significant evidence for neither.

|  | **readabilitys** | | | | **confidences** | | | | **readabilitys** | | | | **confidences** | | | |
| --- | --- | --- | --- | --- | --- | --- | --- | --- | --- | --- | --- | --- | --- | --- | --- | --- |
| *Predictors* | *Estimates* | *CI* | *p* | *df* | *Estimates* | *CI* | *p* | *df* | *Estimates* | *CI* | *p* | *df* | *Estimates* | *CI* | *p* | *df* |
| (Intercept) | 71.70 | 67.03 – 76.38 | **<0.001** | 339.00 | 70.94 | 66.16 – 75.72 | **<0.001** | 339.00 | 70.16 | 60.70 – 79.61 | **<0.001** | 340.00 | 70.21 | 60.15 – 80.27 | **<0.001** | 340.00 |
| difficulty_no | 2.73 | 0.90 – 4.56 | **0.003** | 339.00 | 1.92 | 0.09 – 3.76 | **0.039** | 339.00 | 2.91 | 1.79 – 4.04 | **<0.001** | 340.00 | 1.87 | 0.75 – 2.99 | **0.001** | 340.00 |
| topic [populism] | -14.04 | -20.61 – -7.47 | **<0.001** | 339.00 | -16.06 | -22.48 – -9.63 | **<0.001** | 339.00 | -14.19 | -20.19 – -8.20 | **<0.001** | 340.00 | -15.18 | -21.04 – -9.31 | **<0.001** | 340.00 |
| topic [solar] | -31.61 | -39.32 – -23.90 | **<0.001** | 339.00 | -28.18 | -35.67 – -20.69 | **<0.001** | 339.00 | -32.17 | -38.96 – -25.37 | **<0.001** | 340.00 | -29.71 | -36.31 – -23.11 | **<0.001** | 340.00 |
| difficulty_no * topic [populism] | 0.20 | -2.39 – 2.80 | 0.877 | 339.00 | -0.77 | -3.38 – 1.83 | 0.561 | 339.00 |  |  |  |  |  |  |  |  |
| difficulty_no * topic [solar] | 0.42 | -2.54 – 3.38 | 0.781 | 339.00 | 1.10 | -1.84 – 4.03 | 0.464 | 339.00 |  |  |  |  |  |  |  |  |
| english_first [y] |  |  |  |  |  |  |  |  | 1.91 | -7.38 – 11.21 | 0.686 | 340.00 | 0.76 | -9.27 – 10.80 | 0.882 | 340.00 |
| **Random Effects** | | | | | | | | | | | | | | | | |
| σ^2^ | 598.40 | | | | 541.98 | | | | 592.56 | | | | 544.76 | | | |
| τ_00_ | 72.81 _participant_ID_ | | | | 165.77 _participant_ID_ | | | | 77.24 _participant_ID_ | | | | 163.09 _participant_ID_ | | | |
| ICC | 0.11 | | | | 0.23 | | | | 0.12 | | | | 0.23 | | | |
| N | 170 _participant_ID_ | | | | 170 _participant_ID_ | | | | 170 _participant_ID_ | | | | 170 _participant_ID_ | | | |
| Observations | 347 | | | | 347 | | | | 347 | | | | 347 | | | |
| Marginal R^2^ / Conditional R^2^ | 0.242 / 0.325 | | | | 0.189 / 0.379 | | | | 0.244 / 0.331 | | | | 0.186 / 0.374 | | | |
